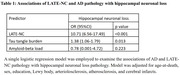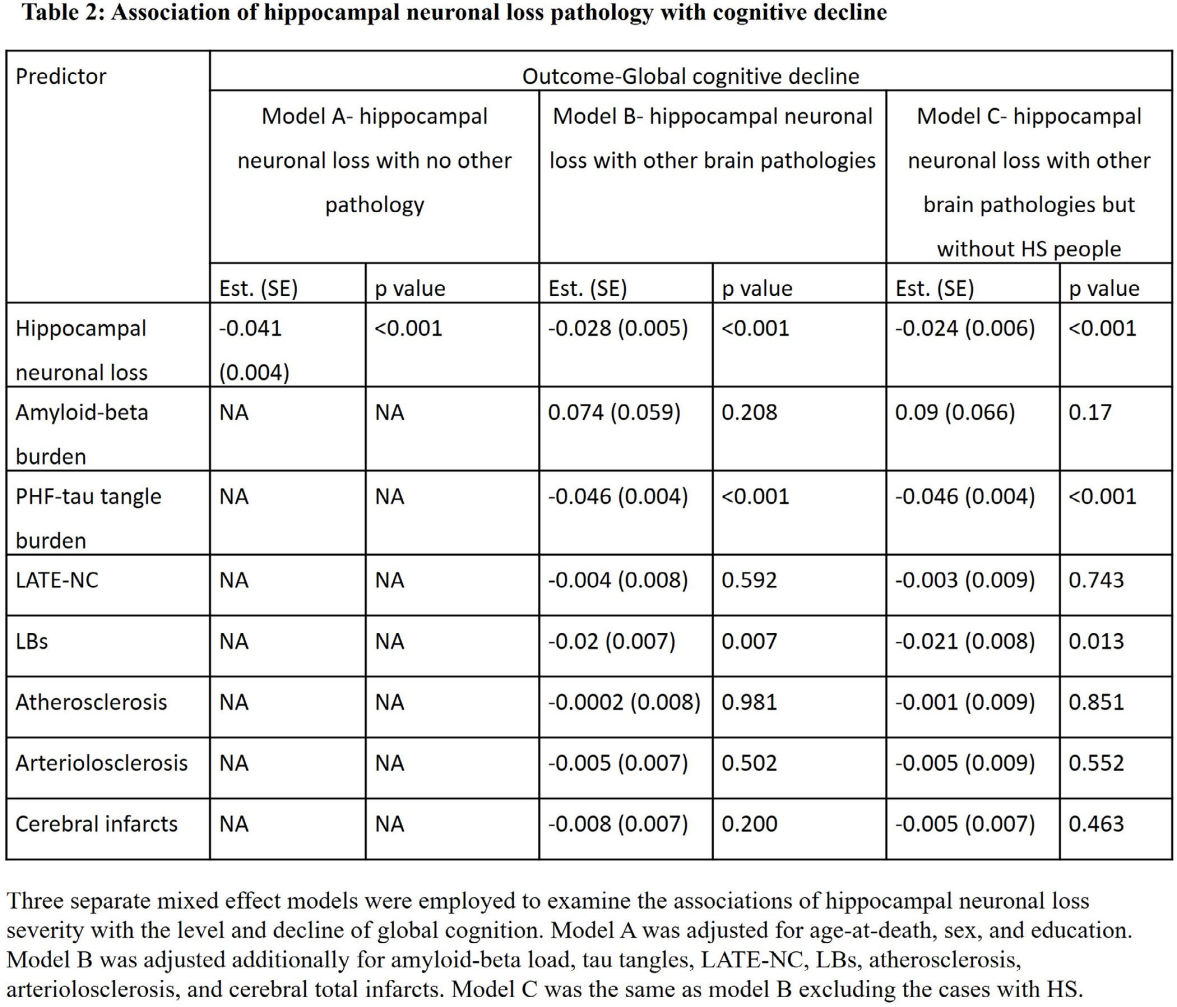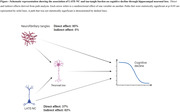# Hippocampal neuronal loss, LATE‐NC, Alzheimer’s disease pathology, and cognitive decline

**DOI:** 10.1002/alz.089069

**Published:** 2025-01-03

**Authors:** Sonal Agrawal, Lei Yu, Sue E. Leurgans, Lisa L. Barnes, David A. Bennett, Julie A. Schneider

**Affiliations:** ^1^ Rush Alzheimer’s Disease Center, Rush University Medical Center, Chicago, IL USA; ^2^ Department of Pathology, Rush University Medical Center, Chicago, IL USA; ^3^ Department of Neurological Sciences, Rush University Medical Center, Chicago, IL USA; ^4^ Department of Psychiatry and Behavioral Sciences, Rush University Medical Center, Chicago, IL USA; ^5^ Department of Neurology and Pathology, Rush University Medical Center, Chicago, IL USA; ^6^ Rush Alzheimer's Disease Center, Rush University Medical Center, Chicago, IL USA

## Abstract

**Background:**

Hippocampal neuronal loss (HNL), LATE neuropathologic changes (LATE‐NC), and Alzheimer’s disease (AD) are common neuropathological findings in older persons. However, the inter‐relationship between AD, LATE‐NC, HNL, and cognition is not well understood.

**Method:**

Participants without known dementia (n = 420; mean age‐at‐death = 92 years, women = 72%) enrolled, in the Rush community‐based cohorts and underwent annual cognitive testing and autopsy. At autopsy, the severity of HNL in the CA1‐subiculum sector was semi‐quantitatively graded from 0 (none) to 5 (severe) on H&E stain without regard to AD or LATE‐NC pathology. Hippocampal sclerosis (HS) was defined as severe hippocampal neuronal loss (grade 5). Tau‐tangle density, β‐amyloid burden, LATE‐NC, and other age‐related pathologies were also recorded. Logistic regression and mixed‐effect models adjusted for demographics and neuropathologies were used to examine the association of HNL with AD (tangles/ β‐amyloid) and LATE‐NC, and separately with cognitive decline. Path analyses examined the extent to which the associations of LATE‐NC and tau tangles with cognitive decline was attributable to HNL.

**Result:**

HNL was common: mild in 61%, moderate in 16%, and severe in 20% of participants. Advanced LATE‐NC stage 2/3 and tau‐tangles, but not β‐amyloid pathology, were associated with worse hippocampal neuronal loss. More HNL was associated with faster decline in global cognition and four cognitive domains: episodic, semantic, and working memory and perceptual speed. These associations remained robust even after excluding participants with HS. LATE‐NC and tangles were also associated with cognitive decline but the association between LATE‐NC and cognitive decline was lost while the association of tangles with cognitive decline was partially attenuated after adding HNL in the model. The result from path anlayses suggested that two‐thirds of the association between LATE‐NC and cognitive decline was attributable to HNL whereas only 5% of the association of tau tangles was through HNL.

**Conclusion:**

Our results support that HNL and tangles are the major contributors of cognitive dysfunction. LATE‐NC is also associated with cognitive decline but may causes most dysfunction via HNL.